# Lignans and Gut Microbiota: An Interplay Revealing Potential Health Implications

**DOI:** 10.3390/molecules25235709

**Published:** 2020-12-03

**Authors:** Alice Senizza, Gabriele Rocchetti, Juana I. Mosele, Vania Patrone, Maria Luisa Callegari, Lorenzo Morelli, Luigi Lucini

**Affiliations:** 1 Department for Sustainable Food Process, Università Cattolica del Sacro Cuore, via Emilia Parmense 84, 29122 Piacenza, Italy; alice.senizza@unicatt.it (A.S.); vania.patrone@unicatt.it (V.P.); marialuisa.callegari@unicatt.it (M.L.C.); lorenzo.morelli@unicatt.it (L.M.); luigi.lucini@unicatt.it (L.L.); 2 Cátedra de Fisicoquímica, Departamento de Química Analítica y Fisicoquímica, Facultad de Farmacia y Bioquímica, Universidad de Buenos Aires, Buenos Aires C1113AAD, Argentina; xuanif@hotmail.com

**Keywords:** phenolic compounds, bioaccessibility, enterolignans, gut microbiota, gut–brain axis

## Abstract

Plant polyphenols are a broad group of bioactive compounds characterized by different chemical and structural properties, low bioavailability, and several in vitro biological activities. Among these compounds, lignans (a non-flavonoid polyphenolic class found in plant foods for human nutrition) have been recently studied as potential modulators of the gut–brain axis. In particular, gut bacterial metabolism is able to convert dietary lignans into therapeutically relevant polyphenols (i.e., enterolignans), such as enterolactone and enterodiol. Enterolignans are characterized by various biologic activities, including tissue-specific estrogen receptor activation, together with anti-inflammatory and apoptotic effects. However, variation in enterolignans production by the gut microbiota is strictly related to both bioaccessibility and bioavailability of lignans through the entire gastrointestinal tract. Therefore, in this review, we summarized the most important dietary source of lignans, exploring the interesting interplay between gut metabolites, gut microbiota, and the so-called gut–brain axis.

## 1. Introduction

In the last years, research efforts have been devoted to the characterization of polyphenols and their metabolites in different biofluids related to potential health-promoting properties [1]. In this regard, a phenolic-rich diet is suggested to provide health benefits for the consumers, mainly when considering protection against oxidative stress and other diseases (Rothwell et al., 2016). Phenolic compounds are classified as plant secondary metabolites, characterized by a chemically diverse group of bioactive compounds and comprising several subclasses like flavonoids, phenolic acids, lignans, stilbenes, and other compounds (such as tyrosol derivatives and tannins, respectively) [2].

Among polyphenols, lignans have been widely studied due to their steroid-analogous chemical structure, being recognized as phytoestrogens, and possessing various biological properties, including anti-inflammatory and antioxidant properties [3,4,5]. Additionally, epidemiological studies observed that lignans decrease the risk of cardiovascular disease [6], whilst other widely studied effect on chronic diseases (such as breast cancer) are still not fully confirmed.

The presence of lignans in the plant kingdom follows a similar pattern of distribution to other phenolic compounds. There are particularly rich dietary sources of lignans, such as oilseeds (flaxseed, sesame, linseed, and sunflower), whilst other foods present moderate to low amounts [7]. Nevertheless, dietary surveys have observed that whole grains (especially rye), legumes, fruits, vegetables, nuts, and some beverages such as tea and coffee, can be considered good dietary sources of these compounds [8,9]. Starting from the previous considerations, it seems clear that the characteristics of the diet will define the degree of exposition to plant lignans. Due to their large occurrence in foods, the most studied compounds are secoisolariciresinol, matairesinol, lariciresinol, pinoresinol, medioresinol, syringaresinol, and sesamin, which are predominantly present as glucosides as secoisolariciresinol diglucoside and pinoresinol diglucoside [7,10,11].

As widely reviewed in the scientific literature [12], lignans originate from cinnamic acid derivatives, which are biochemically related to the metabolism of phenylalanine. In particular, according to the incorporation of oxygen within the pattern of cyclization, lignans can be divided into eight subclasses [3], namely dibenzylbutyrolactol, dibenzocyclooctadiene, dibenzylbutyrolactone, dibenzylbutane, arylnaphthalene, aryltetralin, furan, and furofuran lignans. The nutritional significance of lignans is unknown. Although lignans are not classified as dietary fibers, they share some of the chemical characteristics of lignin (a large plant polymer characterizing the cell wall), which is an insoluble fiber. Overall, the comprehensive database about the phenolic composition of foods, namely, Phenol-Explorer, reports 53 lignans (including both parent compounds and large intestine metabolites). In this regard, the plant lignans most commonly distributed in foods are reported to be lariciresinol, matairesinol, pinoresinol, and secoisolariciresinol. Several other lignans characterize plant foods, including medioresinol (such as sesame seeds, rye, and lemons), syringaresinol (in grains), sesamin, and the lignan precursor sesamolin (in sesame seeds). Other lignans found in foods but not often quantified include arctigenin, cyclolariciresinol (isolariciresinol), 7′-hydroxymatairesinol, and 7-hydroxysecoisolariciresinol. Some cyclolariciresinol occurs naturally and a part arises from lariciresinol during extraction and analysis under acidic conditions. The most common lignan structures and major food sources are presented in Table 1.

Starting from the previous considerations, the present review attempts to describe the potential beneficial effects of lignan intake as related to the modulation of gut microbiota and the possible interactions existing with the so-called “gut–brain” axis. The latter represent a novel topic worthy to be investigated, considering that increasing evidence suggests that food ingested polyphenols can have beneficial effects in neuronal protection, acting against oxidative stress and inflammatory injury.

## 2. Bioaccessibility and Bioavailability of Lignans during Gastrointestinal Digestion and Fermentation Process

The digestion process encompasses concatenated steps, each of them with particular physiological conditions to facilitate the release of food components to the lumen for subsequent intestinal absorption and distribution to organs and tissues.

Under in vitro simulated conditions of the stomach and small intestine, it was observed that release of secoisolariciresinol diglucoside (SDG) from the food digesta depends on the matrix characteristics with no release of secoisolariciresinol (SECO), thus suggesting marginal alteration of lignans during gastric and intestinal steps [10]. Nevertheless, deglycosylation of SDG may occur via the action of brush border enzymes as suggested by the in vivo appearance of SECO in biofluids after the intake of SDG-rich source [11,12,13]. However, the relation among ingested plant lignans and small intestinal-absorbed aglycones is low [14], with a significant proportion reaching the colon for further processing by the local microbiota to produce the enterolignans enterodiol (ED) and enterolactone (EL). Overall, the gut microbial metabolism can be studied through in vitro and in vivo approaches. The most common procedure for in vitro fermentations consists in exposing the plant lignans source to fecal gut microbiota from either human or animal origin. This allows determining the sequence of bacterial reactions involved in the microbial catabolism of lignan precursors by analyzing samples collected at different time points [15]. On the other hand, in vivo studies including dietary interventions (animals or humans) and epidemiological studies are pivotal to know the pharmacokinetic and bioavailability of colonic metabolites and their possible connection with biological activity [9,11,13].

**Figure 1:**
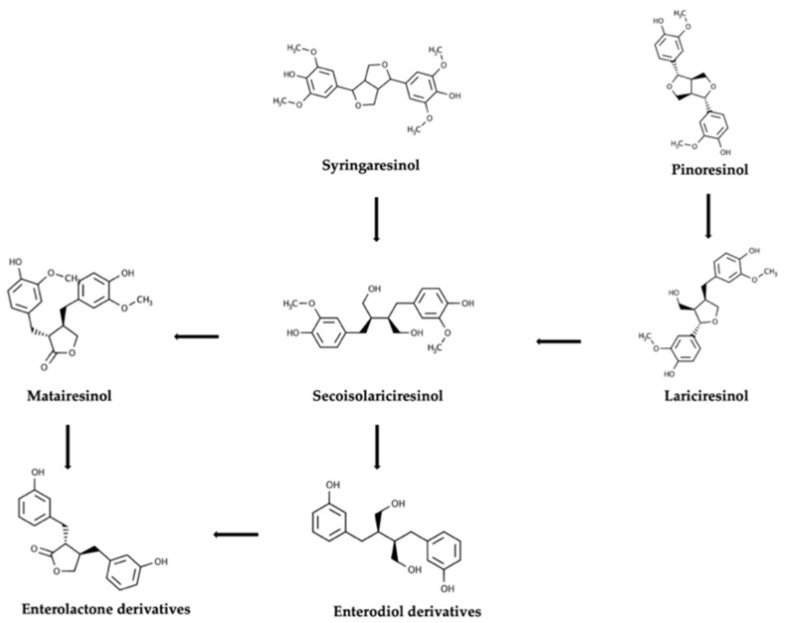
Overview of the colonic pathways involving plant lignans, starting from two parent compounds, namely syringaresinol and pinoresinol.

As exposed previously, the mammalian enterolignans ED and EL are the common major microbial end metabolites of plant lignans [15,16]. Hence, the colonic pathways of plant lignans can be almost wholly described taking as the starting point the parent compound further transformed to simpler compounds to conclude in the generation of ED and EL as promoted by microbial phase I reactions [15,16,17]. An overview of the proposed colonic transformation pathway of plant lignans is reported in Figure 1.

Overall, pinoresinol diglucoside (PDG) undergoes two sequential deglycosylations to initially produce pinoresinol (PINO) monoglycoside and then PINO aglycone, which is converted to lariciresinol (LAR) by means of reduction reactions [18]. Demethylation of LAR produces demethyl-LAR, which by subsequent reduction generates dimethyl-SECO; nevertheless, this route has been observed to possess little relevance [18]. Alternatively, the reduction of LAR produces SECO that it can also be originated by deglycosylation of non-absorbed dietary SDG [15]. In addition, LAR and SECO were identified in the urine of rats received a daily oral gavage of 15 mg of sesamin/kg b.w. during 10 d indicating they can also be originated by consecutive bacteria demethylation and dehydroxylation of sesamin [19].

In view of the above, the final and decisive stages of the gut metabolism of many of the most common plant lignans can be described by bacterial transformations of SECO. The analysis of available data from in vitro and in vivo studies allows us to construct a detailed description of the colonic fate of SECO. In this regard, luminal SECO can be originated by microbial metabolism of plant lignans precursors or by non-absorbed SDG. Additionally, as reported by several in vitro studies, SDG is converted to SECO after two successive deglycosylations, leading to the transient appearance of SECO monoglycoside (SMG) [15,17]. Deglycosylation was observed to be a common property of a wide range of gut bacteria but specified consortia have the capability to continue metabolizing SECO [16,19]. Accordingly, a total of 17 metabolites, including intermediates (i.e., ED and EL), have been related to the colonic metabolism of SECO [15,17]. Initial demethylation of SECO generates demethyl-SECO from which demethyl-dehydroxy-SECO, didemethyl-SECO (dihydroxy-ED), didemethyl-dehydroxy-SECO (hydroxy-ED), and ED are generated by a combination of demethylation and dihydroxylation reactions [15]. It was proposed that the demethylation of SECO should activate subsequent SECO modifications [20]. An important metabolic step is the oxidation of ED, after which EL is generated. Another described route for the production of EL involves the generation of MAT after dehydrogenation of SECO. Nevertheless, MAT not always was successfully detected as an intermediate product of SECO [15,17] but the appearance of related derivatives as intermediates did not reject this hypothesis. These lactones derivatives have been described as demethyl-MAT, demethyl-dehydroxy-MAT, didemethyl-MAT (dihydroxy-EL), and didemethyl-dehydroxy-MAT (hydroxy-EL) although a second alternative for the generation of these metabolites was associated with dehydrogenation of the metabolites described previously [15,18]. On the other hand, both MAT and hydroxy-MAT (HMAT) are provided by several food products, making them an important dietary source of EL and hydroxy-EL precursors. In the colonic pathway of SECO cyclic ethers derivatives were also described, in which the first reaction involved in the appearance of anhydrosecoisolariciresinol (AHS) remains still to be clarified [15]. The anhydrous metabolites were described after the in vitro fermentation of SDG [15] and synthetically obtained AHS [21] and include demethyl-anhydro SECO, didemethyl-anhydro SECO, and didemethyl-dehydroxy-anhydro concluding with the possible formation of enterofuran [15,21]. The colonic conversion of plant lignans into each other and the identification of some of the above-described intermediate metabolites were also observed in urine, plasma and/or feces after acute or sustained intake of dietary lignans in humans [9,15] and animals [21], which validate in vitro fermentation models to study the colonic fate of plant lignans.

## 3. Intraindividual and Interindividual Variability in the Conversion of Plant Lignans

In an attempt to explore the gut transformation of plant lignans into enterolignans, both intraindividual and interindividual differences in the conversion rate, maximal yield, and pattern profile need to be carefully considered. Diverse intraindividual metabolic capacities are mainly associated with the chemical structure of the precursor and/or the matrix characteristics of the plant lignan source. However, exactly defining the effectiveness of such conversion, according to the type of lignan molecule, is a difficult task due to the inconsistency of results. Nonetheless, both in vitro [19,22] and in vivo [23] studies reported that the catabolic transformation of SECO and MAT was observed to be greater than LAR, SYR, PINO, and sesamin and, in turn, that aglycones are more efficiently metabolized than diglucosylated derivatives. Except for MAT that generates EL but not ED, the contribution of each plant lignan to the whole pool of ED and EL is not entirely clear [19]. Smeds et al. [24] have observed that after a sustained oral dose of SECO and LAR to rats for 10 d, the ED:EL ratio decreased compared with the first single dose indicating that probably an adaptation of gut microbiota occurs during the dosage period toward a major efficiency to convert EL from ED. Nevertheless, the ED:EL ratio seems to be more closely associated with gut microbiota activity than the lignan molecule. Furthermore, the role of the food matrix and the origin source of plant lignan should not be underestimated since they may also explain differences, particularly in the production yield of enterolignans [10].

Wide interindividual variability in the magnitude range and profile of enterolactones is a common observation in experimental and observational studies. Idiosyncrasy explains part of the individual physiological responses, but other aspects could contribute to understanding these deviations. Dietary interventions in which plant lignan-rich products were provided in a single [11] or sustained dose [9,24,25,26] result in an evident increase of excreted and circulating levels of enterolignans. Observational studies also support these findings since variable amounts of enterolignans were quantified in the urine of healthy women (0.52, 1.95, and 6.22 ug/mg creatinine for low, middle, and high enterolactone excreters, respectively) [5] and men (1.57 and 5.96 µmol/24 h for low and high enterolactone excreters, respectively) [27] and in plasma of endoscopy individuals (4.5, 15.2, and 40.1 nmol/L for low, middle, and high enterolactone producers, respectively) [28] and, in both cases, the highest tertile corresponded with those volunteers who reported major intake of plant lignans-rich food in habitual diet, especially dietary fiber.

Along with diet, the capacity of gut microbiota to metabolize non absorbed plant lignans determine the coexistence of different phenotypes: low, middle, and high enterolignan producers [8,9,29], although other factors including genetics [30], sex [31], and age [20,32] could also contribute to metabolic differences. It was suggested that microbial dehydrogenation of ED to generate EL is a crucial step in the colon metabolism of plant lignans that could explain major variation in enterolignan production [33]. The shift toward a major production of EL is desirable because this metabolite showed a stronger association with health benefits than ED [34]. Eeckhaut et al. [29] carried out an in vitro fermentation of flaxseed extract rich in SDG using feces from low and high enterolignan producers and observed that the later donor produced ten times more EL (200 nM) than the former (20 nM) whereas the amount of ED was similar in both batch cultures. This exciting finding was partially reproduced in humans after single or sustained supplementation of plant lignans. After the acute intake of SDG by healthy adults of both sexes, the cumulative excretion of ED and EL was 966 ± 639 nmol/L·h for and 1762 ± 1117 nmol/L·h, respectively. Among the participants, it was determined that EL was more than twice than ED in 5 out of 12 subjects, between 1 and 2 times in 5 out of 12 subjects and in 2 volunteers excreted ED was higher than EL [31]. In another study, after the daily intake of 0.3 g flaxseed/kg body weight by healthy man for 1 week, the mean serum concentration of ED and EL increased 10-fold compared to baseline until reach 20 and 60 nM, respectively, but not all the participant responded to the same form since ED increased in all participants while EL remained unchanged in two subjects [26]. Many dietary interventions including pharmacokinetic studies in human [11] and animals [23,24] and prolonger plant lignan supplementation in human [13] have presented the mean values of ED and EL or only EL in biofluids before and after the intervention. Still, they have not discriminated among categories of different metabolic responses losing the opportunity to gather data to define how individuals respond to plant lignans supplementation as proposed by other authors [15].

Overall, evidence that suggests that the production pattern of EL vary within age exists. For example, in an in vitro fermentation of the oilseed mix it was observed that the fecal microbiota from healthy young women generates more EL than those from premenopausal donors and this difference was attributed to diverse intestinal microbiota profile [20]. This observation could explain a previous study in which a lower proportion of feces from postmenopausal women could generate EL compared with ED following in vitro fermentation of flaxseed extract [35]. These results were not exactly reproduced under in vivo conditions. Postmenopausal women received an acute administration of 86 and 172 mg SDG showed major urinary EL (46.93 and 80.45 µmol/96 h for the low and high dose, respectively) than ED (29.78 and 46.3 µmol/96 h for the low and high dose, respectively). On the other hand, Milder et al. [28] have observed an association between older volunteers and major circulating ED and EL. Nevertheless, the ED:EL ratio in urine was lower in younger than in older volunteers [10]. In addition, the reduced plant lignans metabolic capacity is not only exclusive to old women, but also involves young children [32] and man [31]. Feces from children lower than 3 years-old inoculated with SECO generate dihydroxienterodiol but not ED and only 9 out of 24 fecal samples were able to generate EL, thus suggesting the presence of immature microbiota lacking in a bacteria population able to participate in some essential metabolic steps for the generation of mammalian enterolignans [16]. The differences attributable to sex were noticed after a pharmacokinetic study where the plasma peak of EL appear earlier in women than men and the plasma concentration of EL and tended to be higher in women, but not differences were found in urine. Additionally, the differences attributable to sex were noticed during a pharmacokinetic study where EL peaked earlier and tended to be higher in women than men, although no significant differences were found in urine [28].

In summary, alternative and simultaneous branches of metabolic pathways to transform non absorbed plant lignans into enterolignans do exist. These pathways are mainly defined by bacterial demethylation, reduction, dehydroxylation, and dehydrogenation; nevertheless, the main final metabolites are ED and EL. A comprehensive list of the most interesting studies can be observed in Table 2. However, beside the intrinsic characteristics of individual lignan molecule, diet, and gut microbiota (conformation and activity) are dominant factors affecting the amount and profile of mammalian enterolactones produced in the colon. Therefore, in the context of plant lignans colon metabolism it was observed great interindividual differences, which has led to the identification of different phenotypes, which can be affected by age and sex, according to the capacity to produce enterolignan.

## 4. Modulation of Gut Microbiota by Enterolignans

Diet represents one of the main factors that can modulate the bacterial richness and abundance in the gastrointestinal tract of humans and animals [42]. In this regard, the metabolites released by gut microbiota influence the physiological activities of the host [43]. Furthermore, intestinal bacteria provide short chain fatty acids (SCFAs) and other metabolites as a result of diet component degradation [44]. All these metabolites exert effects on both the host and other bacteria within the intestinal microbial community. They act as signaling molecules or as substrates for subsequent metabolic reactions. One of the most investigated fields of research is the fiber impact on gut microbiota metabolisms. In particular, plant-based diet rich in fiber have been linked to a *Prevotella* enterotype, whereas the *Bacteroides* enterotype was associated with a high intake of protein and fat [45,46]. Of particular interest are the results from an in vivo study, when considering the Hazda population and children from Burkina Faso [47,48]. In fact, the gut microbiota of Hazda population was enriched in *Prevotella*, *Treponema,* and an unclassified *Bacteroidetes*, while the diets of Burkina Faso children showed high abundance of *Prevotella* and *Xylanibacter* species. In both cases, diet was primarily plant-based and this favored groups of bacteria particularly efficient in degrading complex carbohydrates. The digestion of dietary fiber requires specific bacterial pathways due to the high content of complex polysaccharides and other compounds such as polyphenols, mainly tannins and lignans, which negatively affect their degradation. As widely discussed, polyphenols are usually classified into different subfamilies, according to their chemical structure. Their scaffolds represent the specific characteristic of the molecule and at the same time is the limiting factor for the production of possible final bioactive products. In fact, only some structures can be biotransformed by enzymes of both host and gut microbiota.

Regarding the transformation of lignans into enterolignans (i.e., enterodiols and enterolactones), despite the huge number of bacteria inhabiting gut microbiota, only few species have been identified as involved in such conversion [20]. Besides, the enzymes responsible for this transformation remain unknown [49]. Although mucosal enzymatic activities and chemical hydrolysis in the stomach may influence the bioavailability of lignans, the mechanisms underlying these processes are still under investigation. Indeed, controversial results are available in literature, since Clavel et al. [50] reported that SDG was not hydrolyzed after prolonged incubation at 37 °C in artificial stomach juice (in vitro study). These results were in agreement with other similar experiments, including other lignans substrates [51]. Thus, this data suggested that the bioavailability of SDG and other glycosylated lignans is not fully related to the hydrolysis promoted by the stomach juice, as normally described. Conversely, their bioavailability is strictly associated with gut microbiota metabolism. Hence, the role of gut microbiota in converting these compounds is very relevant since intestinal bacteria can turn some plant lignans such as sunflower, flaxseed, caraway, legumes, pumpkin, and soybean [52], into mammalian lignans.

Several biochemical steps are involved in plant lignan transformation into enterolignans and the consortia of bacteria share metabolic intermediates. For example, bacteria utilize four sequential reactions to convert SDG to enterolactone (EL): O-deglycosylation, O-demethylation, dehydroxylation, and dehydrogenation [53]. Despite the in vitro nature of the approach used, interesting conclusions concerning host-related metabolic capability were reported in literature. In fact, SDG was completely metabolized using human fecal microbiota, whereas the last step of this reaction did not occur when rat fecal inoculum was used. Of particular interest, a possible pathway involved in SDG metabolization was reported. This transformation was a cascade process that led to eight different compounds, corresponding to seven different enzymatic reactions. The role of enzymes in these conversions was confirmed by the absence of SDG degradation products in negative control without bacterial inoculum. Clavel et al. [50] demonstrated that the first reaction involving the O-deglycosylase was closely related to the genera of *Bacteroides* and *Clostridium*. Among these, *Clostridium cocleatum*, *Clostridium ramosum*, *Clostridium saccharogumia*, *Bacteroides distasonis*, *Bacteroides fragilis*, and *Bacteroides ovatus* species have been demonstrated to catalyze this first reaction.

The second reaction in the conversion of SDG to EL is the O-demethylation of secoisolariciresinol (SECO) [54] through the intermediate 2,3-bis-(3,4-dihydroxy-benzyl)butane-1,4-diol. *Eubacterium limosum* and *Blautia producta* and other bacteria are able to catalyze this conversion [50,53]. The O-demethylases proteins purified from strains of *Eubacterium limosum*, *Moorella thermoacetica*, *Acetobacterium dehalogenans,* and *Desulfitobacterium hafniense* have been investigated for their properties [54,55,56,57]. According to these studies, O-demethylase is composed of four components with a specific function and three of them are directly involved in the SECO conversion, whereas the fourth plays an accessory role. In fact, this protein reduces the corrinoid proteins, resulting by the enzymatic SECO conversion, thus reactivating them. Conversely, the other three enzymes are involved in a cascade process as described by Chen et al. [58]. In particular, genes encoding for the first three components are organized in an operon, thus suggesting their simultaneous transcription, whereas the last one is separated from this locus. This indicates that the component responsible of the corrinoid protein reduction can act separately from the other three subunits, likely reactivating similar proteins in other enzymatic reactions [58].

The third step is the dihydroxylation step to convert matairesinol into enterodiol (ED). ED is rapidly converted into EL via dehydrogenation. Consistent with this hypothesis, in a human trial, ED was detected only in five subjects out of 14, while EL was found in all individuals [59].

Despite the information previously reported, no microorganisms have been identified that can completely metabolize the plant lignan SDG to EL. Thus, in a complex microbial community, cooperation between different groups of bacteria led to the production of EL. As an example, *Eggerthella lenta* is not able to directly convert SECO to EL, since it specifically converts an intermediate compound, the 2,3-bis-(3,4-dihydroxy-benzyl)butane-1,4-diol, to this final product. The entire conversion can be conducted by *E. lenta* when it is cocultured with *Blautia producta*. Indeed, this last species is able to produce the aforementioned intermediate that can be used as a substrate by *E. lenta*. We could speculate that such a relationship proved using an “in vitro” study could explain the production of EL also in the intestinal environment.

Concerning in vivo studies, little information is available in scientific literature. Of interest, in gnobiotic rats, the infection with *Clostridium saccharogumia*, *E. lenta*, *B. producta*, and *Lactonifactor longoviformis* and the administration of plant lignans SECO allow one to investigate the production of EL and ED. In the urine of these rats, ED and EL were detected, but not SECO. On the contrary, in the urine of not colonized animals, SECO was found but not its bioactive enterolignans [60]. These observations confirm the role of bacteria in SECO conversion and the “in vitro” studies. In human, gut microbiome composition of young healthy (25–30 years) and premenopausal women was assessed after incubation in batch in the presence of an oilseed mix. The ENL production increased in young women, whereas an increase of ED was detected in premenopausal subjects. Differences in *Clostridiaceae*, *Klebsiella* sp. and *Collinsella* sp. were observed. In particular, *Clostridiaceae* appeared higher in premenopausal volunteers than in the younger, thus suggesting the role of this family in lignans degradation.

Overall, it is important to highlight the in vitro nature of the most important findings in this field, thus representing a strong limitation when translated to in vivo applications. In fact, several data obtained did not consider the complexity of bacterial community and intestinal environment conditions. The conversion of lignans to enterolignans is influenced by many factors, such as pH, oxygen pressure, and temperature. On the other hand, these kinds of studies represent the best approach to investigate bacterial behavior without interference from host and other bacterial metabolisms. Therefore, many bacteria that are known to convert lignans into enterolignans have low abundance in the human intestine, thus making it difficult the study through an in vivo approach.

## 5. Potential of Enterolignans as Health-Promoters and Modulators of the Gut–Brain Axis

In the last years, several research works demonstrated that dietary polyphenols could have beneficial effects in cognitive functions, by acting against oxidative stress and inflammatory injury [61,62,63]. Overall, biotransformation of polyphenols is a pivotal step to obtain metabolites active in brain, through a set of microbiota-related reactions occurring in the gut [63]. In this regard, evidence suggests that polyphenols exert beneficial effects acting through multiple pathways involved in oxidative/inflammatory stress signaling and leading to the expression of antioxidant enzymes neurotrophic factors, and cytoprotective proteins. All these processes contribute to maintaining brain homeostasis [64]. Epidemiological studies reported a strong protective effect of a lignan-rich diet towards cardiovascular diseases [65]. The structural similarity between enterolignans and 17β-estradiol allows enterolignans to represent natural ligands of estrogen receptors [66]. Consistently, a high lignan intake has been associated with beneficial effects on human health, due to reduced cardiovascular risk that involves the lowering of LDL cholesterol [67] and the modulation of lipid metabolism [68]. Despite showing pro-oxidant potential at high concentrations and in the presence of transition metal ions (like the other phenolics do), lignans have been also described as strong antioxidants via both a direct radical scavenging activity and an indirect enhancement of the activity of antioxidant enzymes [69]. The enhanced antioxidant capacity imposed by lignans results in the regulation of key molecules involved in inflammation, resulting in the decrease of proinflammatory cytokines and mediators [69].

It is known that oxidative stress is closely related to neuroinflammation phenomena, and that both processes are typically involved in neurodegenerative disorders. Phenolic compounds have been demonstrated to prevent neurological disorders through modulation of the gut–brain axis [70]. A recent study [61] using different neuronal systems, reported that metabolites from dietary polyphenols exert neuroprotective effects after reaching the brain by crossing the blood–brain barrier. Additionally, it was suggested that mammalian enterolignans play a prominent role, even higher than their precursors, against neurodegeneration [71]. That is why considerable research effort was devoted to a complete understanding of the colonic transformations of plant lignans, leading to the generation of ED and EL, as explained in the previous sections. Polyphenols indirect actions involve mechanisms that improve the peripheral cerebrovascular health. Several studies in humans indicated that dietary polyphenols improve vasodilatory response and increase levels of circulating nitric oxide (NO) species that are essential in the control of vascular tone, vasodilation and blood flow in the body and in cerebral circulation [72,73].

**Figure 2:**
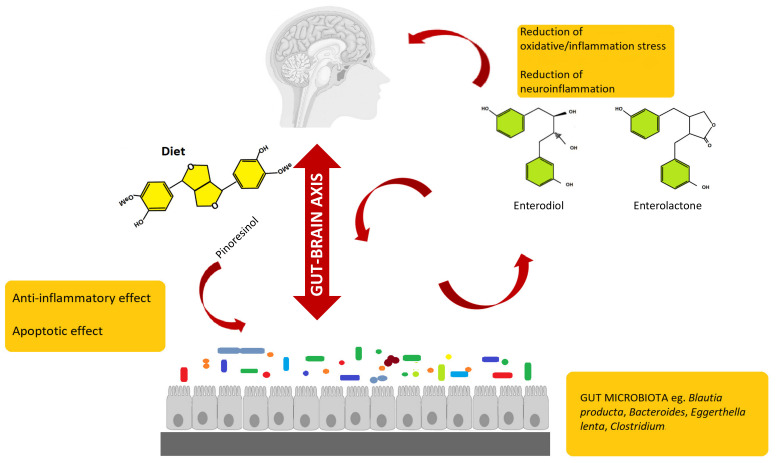
Schematic representation of the gut microbiota–brain interaction, when considering dietary lignans and their gut metabolites (enterodiol and enterolactone). Besides, the main bioactive properties of both parent compounds and gut metabolites are also reported.

Gut microbiota can interact with a central nervous system through different mechanisms and pathways, such as the neurosystems implicated in stress and stress-related disorders (sympathetic and parasympathetic branches of the autonomic nervous system and neuroendocrine and neuroimmune systems) [74,75,76,77]. The communication between these pathways occurs through the vagus nerve, specific metabolites, neurotransmitters, and brain neurotrophic factors [78,79,80,81]. Gut microbiota is able to synthetize neurotransmitters, and thus microbiota homeostasis can impact on complex neurodegenerative disorders. Examples are short-chain fatty acids (SCFAs), tryptophan, GABA, and a brain-derived neurotrophic factor (BDNF) [82]. Specifically, the connection between gut microbiota and the central nervous system, the so-called gut–brain axis, plays an important role in stress response and it is widely recognized as the neuroendocrine system [83,84,85,86,87]. Communication between gut and brain involves multiple overlapping pathways: the enteric nervous system and the neuroimmune and the neuroendocrine systems [88,89]. By interacting with the nervous, endocrine, and immune systems, gut microbiota can influence both directly and indirectly the brain functions. A schematic representation of the gut microbiota–brain interaction when considering lignans and their metabolites is provided as Figure 2.

Overall, the microbiota directly influences the brain functions via the production of neurotransmitters and neuropeptides, in turn acting on neuroactive metabolites production [90,91,92]. In fact, some of these neurotransmitters are able to cross the mucosal layer of the intestines, thus reaching the brain where they can mediate different physiological events [93]. Alteration in the homeostasis of gut–brain axis has been associated also to neurological disorders and neurodegenerative diseases [94]. Moreover, it is emerging that the regulation of microbiota composition can be realized using natural bioactive molecules such as polyphenols derived by plants, suggesting that polyphenols could be used to restore the altered brain functions that characterize neurodegenerative diseases.

Flaxseed-derived lignans, secoisolariciresinol diglucoside (SDG) and pinoresinol diglucoside, are metabolized by the intestinal bacteria, *Ruminococcus* species, in humans, to give the enterolignans (+)-dihydroxyenterodiol and (+)-enterolactone [95,96]. This gut bacterial species accomplishes both deglycosylation and demethylation of plant-based lignans to give human lignans that are of broad physiological effects. Enterolactone has been related to anticancer activity [97,98]. For example, it was shown that enterolactone inhibits the growth of prostate cancer cell lines in vitro and in vivo, through a caspase-dependent pathway [99]. Enterolactone and secoisolariciresinol are inhibitors of carbonic anhydrase [100] and acetylcholinesterase and butyrylcholinesterase, and thus may afford neuroprotection [101]. In this regard, Alzheimer’s disease (AD) patients show lower levels of acetylcholine in the neuronal synapses, thus leading to the memory loss phenomena. Therefore, through the inhibition of the acetylcholinesterase, these lignans may prevent memory loss in AD patients, hence providing a nutritional strategy to complement the use of synthetically derived acetylcholinesterase inhibitors. In fact, the available commercial pharmaceuticals (i.e., donepezil, galantamine, and rivastigmine) only show a symptomatic relief, whereas lignans may, in addition, help in attenuating the progress of AD [102]. As a further example, the gut bacterial metabolite enterolactone, formed through intestinal bacterial metabolism of the lignan 7-hydroxymatairesinol (HMR), was found to attenuate the degeneration of the striatal dopaminergic terminals in Parkinson’s disease (PD), in the PD rat models [103].

Therefore, dietary polyphenolic compounds are potentially able to affect the gut–brain axis via modulation of the gut microbiota, and thus may be used as nutraceuticals in the prevention of neurological disorders. However, with this regard, it is important to bear in mind that the role of gut microbiota in the health outcomes of dietary (poly)phenols is of paramount importance [104].

## 6. Conclusions and Future Perspectives

Polyphenols may be useful for chronic intestinal disorders, acting at the intestinal level where they achieve their highest concentration in the human body, interacting with inflammation-related cellular signaling pathways and modulating gut microbiota. Furthermore, they can also provide an interesting strategy to defeat neurological disorders, either by direct mechanisms, involving the reduction of neuroinflammation and the enhancement of memory and cognitive functions, or by indirect mechanisms, implying the modulation of gut microbiota and the reduction of intestinal inflammation. Interestingly, native lignans, despite exerting health benefits in the gastrointestinal tract, are substrates for gut microbiota-derived metabolites that are involved in the systemic effects attributed to their parental compounds. Dietary lignans and their gut metabolites should, therefore, be considered as promising nutraceuticals for preventing chronic disorders. In particular, the regulation of gut microbiota composition using polyphenols (such as lignans) may help to restore gut equilibrium and to set up new therapeutic intervention in neuropathologies. In fact, since brain dysfunctions can be linked with dysbiosis of the gut microbiota, a rebalance in the gut microbiota composition may result in a partial or complete reversion of the diseases. Finally, regarding future research directions, a better understanding of the interplay existing between lignans (mainly when considering the gut metabolites enterolignans) and gut microbiota will provide more insight into their health effects, thus opening new possibilities to develop microbiota-based therapies for treating neuronal disorders.

## Figures and Tables

**Table 1:** Most common lignans in plant foods, together with their structure, class, and major sources, according to the comprehensive Phenol-Explorer database [4].

Compound	Structure	Class	Major Food Sources
Secoisolariciresinol	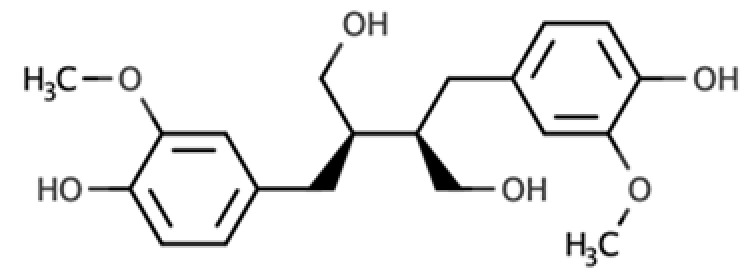	Dibenzylbutane lignan	Flaxseed (257.6 mg/100 g FW) Cashew nut (6.7 mg/100 g FW)
Matairesinol	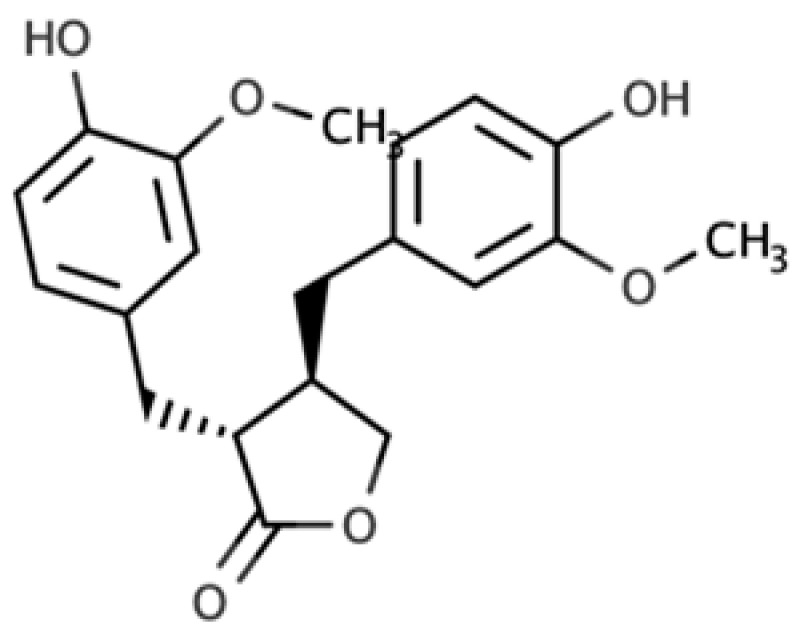	Dibenzylbutyrolactone lignan	Sesame seed (29.8 mg/100 FW) Flaxseed (6.7 mg/100 g FW)
Lariciresinol	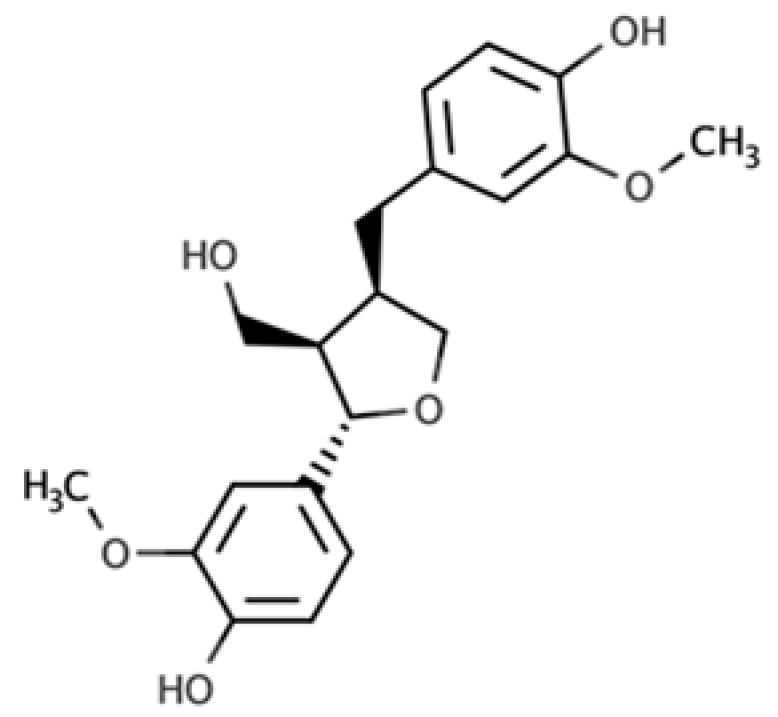	Furan lignan	Broccoli (97.2 mg/100 g FW) Kale (59.9 mg/100 g FW)
Medioresinol	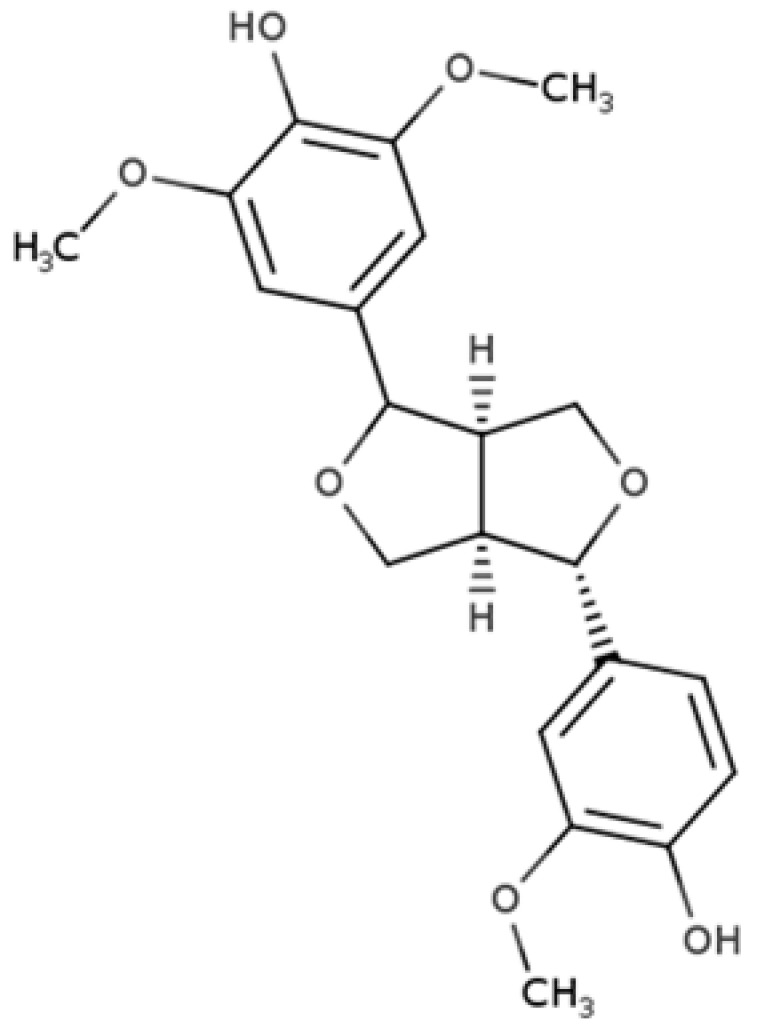	Furofuran lignan	Sesame seed (4.1 mg/100 g FW) Cloudberry (0.48 mg/100 g FW)
Pinoresinol	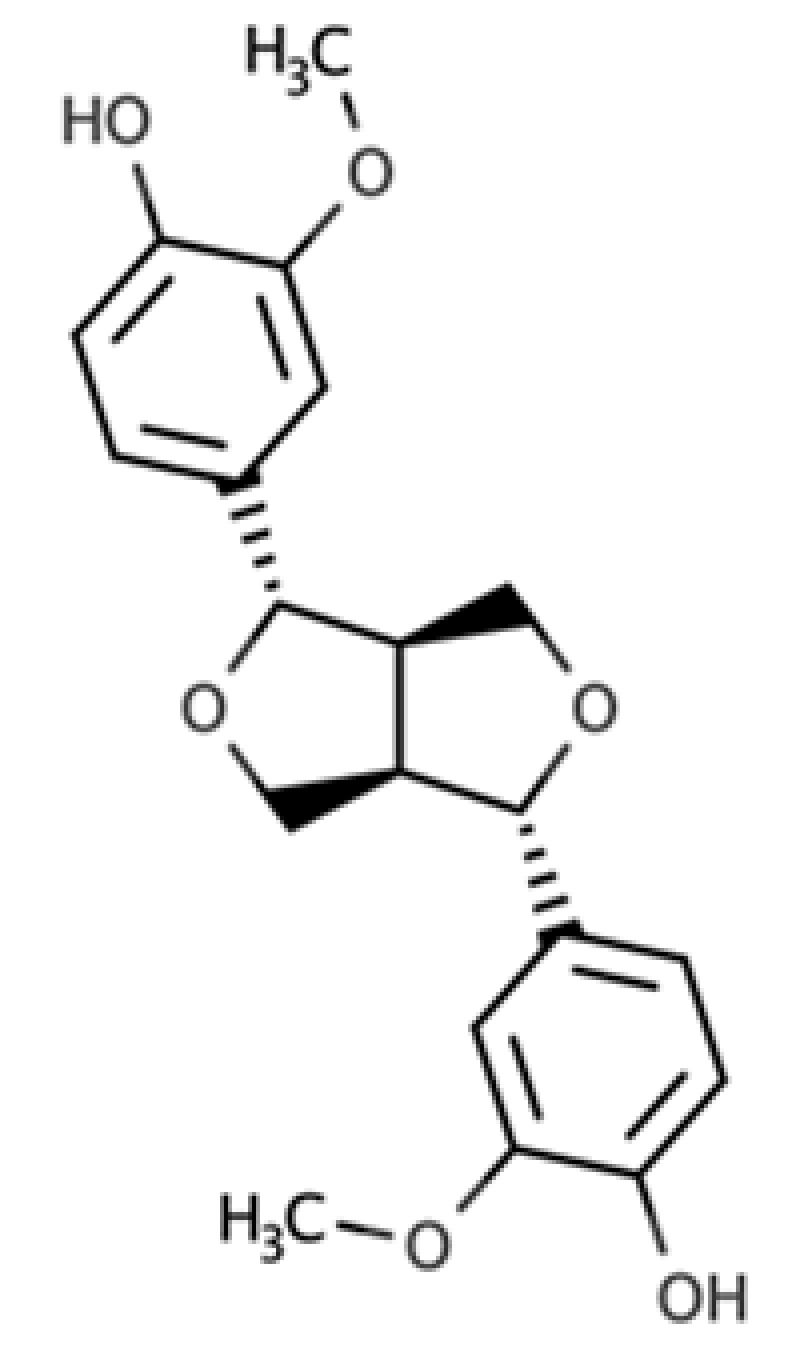	Furofuran lignan	Olive oil (2.4 mg/100 g FW) EVOO (0.42 mg/100 g FW)
Syringaresinol	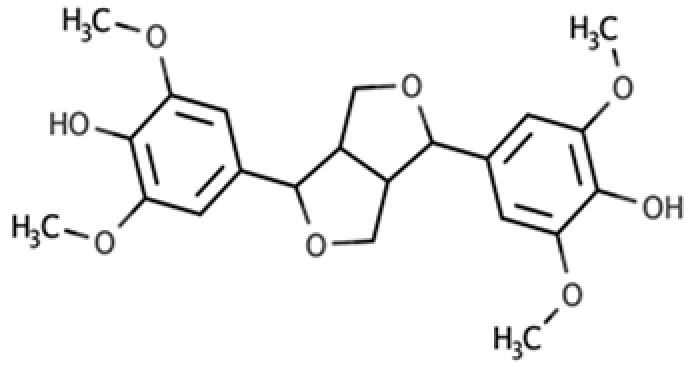	Furofuran lignan	Rye, whole grain flour (0.9 mg/100 g FW) Avocado (0.4 mg/100 g FW)
Sesamin	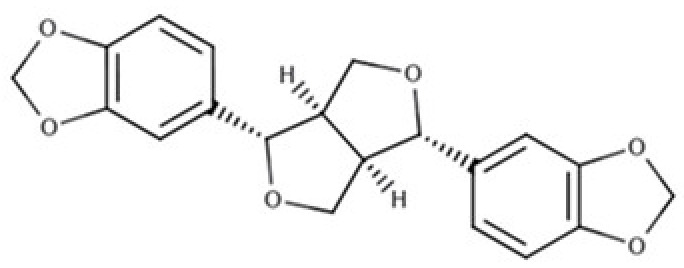	Furofuran lignan	Sesame seed, oil (644.5 mg/100 g FW) Sesame seed (538.1 mg/100 g FW)
Sesamolin	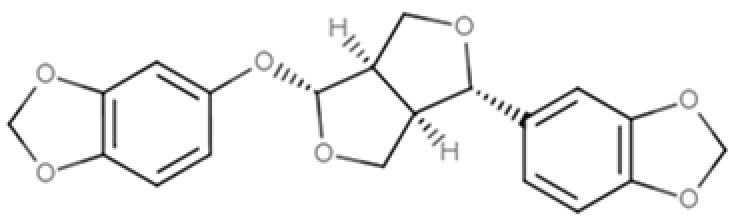	Furofuran lignan	Sesame seed, oil (287.3 mg/100 g FW) Sesame seed (133.9 mg/100 g FW)

**Table 2:** Studies summarizing the production of lignan metabolites (enterodiol and enterolactone) following the intake of lignans from different plant foods.

Plant Lignan Source	Experiment Type	Main Findings	Reference
Wheat bread; Rye bread	Crossover intervention using pigs.	Conversion of parent lignans in EL. MAT and SECO showed the higher conversion rate.	[36]
Oilseeds mix	In vitro fermentation using women feces.	Lower effectiveness in the conversion ED in EL in postmenopausal period.	[20]
BeneFlax^®^	In vivo study based on healthy adults.	Plasma concentration of flaxseed lignans SECO, ED, and EL correlated with daily oral supplementation of flaxseed lignan–enriched complex.	[13]
LinumLife Extra	SHIME (considering low and high enterolignan producers)	Marked differences in EL/ED ratio over the experimental period.	[29]
Whole flaxseed and flaxseed flour	In vitro fermentation using pooled human feces.	Major ED production as resulting by flaxseed flour.Similar production of EL for both food matrices.	[10]
Flaxseed extract	In vitro fermentation using children feces.	Dihydroxy-ED detected as the major metabolites.	[32]
Single lignan compounds (MAT, SECO, PDG, SYR diglucoside, HMAT).	In vitro fermentation using pooled human feces.	Major conversion rates observed for SECO (72%), MAT (62%), and PDG (55%).	[37]
Habitual diet	Food record on urine collected from premenopausal women.	ED was detected over the limit of detection as major metabolite in all urine samples.	[38]
Ground linseed	Healthy women increasing the consumption of fruit and vegetables. Collection of serum and urine samples.	Increase in the concentration of ED (as main metabolite) in both serum and urine samples.	[39]
Isolate SDG	Plasma and urine samples collected from mean and women.	Extraction of urinary EL was 2-fold higher than enterodiol. Plasma concentration of ED was higher in women.	[31]
Wheat and rye diet	Feces and urine collected from pigs.	EL was the predominant circulating lignan found in both biofluids and significantly correlated to the higher plant lignan intake.	[25]
Flaxseed	Collection of serum samples from healthy men.	10-fold increase in serum concentration of ED and EL.	[33]
Sesame, flaxseed and sesame seeds	In vitro fermentation and collection of urine from rats.	Higher conversion rate of parent compounds in ED.	[19]
Habitual diet	Food record on endoscopy men and women (collection of plasma samples).	Higher levels of EL detected in biofluids.	[28]
Whole grains and refined grains	Food record on urine samples collected from health volunteers.	No linear correlation between lignans intake and EL excretion.	[9]
Habitual diet	Food record on urine samples collected from healthy men.	EL production was correlated with a higher intake of vegetables and berries consumption.	[27]
Pure sesamin and sesame seed	In vitro fermentation using pooled human feces.	EL was the main metabolite of sesamin.	[40]
LinumLife^TM^	In vitro fermentation using feces collected from women.	ED detected in higher % when compared with EL. High inter-variability detected.	[35]
SDG and flaxseed consumption	In vitro fermentation and collection of urine samples.	Great inter- and intra-variability detected when considering the different donors.	[15]
Single compounds (SECO, HMAT, and MAT)	Urine samples collected from rats.	Different proportions of excreted EL and ED depending on the lignan precursor.	[41]
Flaxseed extracts (high in SDG)	Serum and urine samples collected from postmenopausal women.	Great dose-response effect observed when considering EL and ED production.	[11]

## References

[1] Rothwell J.A., Urpi-Sarda M., Boto-Ordoñez M., Llorach R., Farran-Codina A., Barupal D.K., Neveu V., Manach C., Andres-Lacueva C., Scalbert A. (2015). Systematic analysis of the polyphenol metabolome using the Phenol-Explorer database. Mol. Nutr. Food Res..

[2] Giuberti G., Rocchetti G., Lucini L. (2020). Interactions between phenolic compounds, amylolytic enzymes and starch: An updated overview. Curr. Opin. Food Sci..

[3] Rodríguez-García C., Sánchez-Quesada C., Toledo E., Delgado-Rodríguez M., Gaforio J.J. (2019). Naturally lignan-rich foods: A dietary tool for health promotion?. Molecules.

[4] Rietjens I.M.C.M., Louisse J., Beekmann K. (2017). The potential health effects of dietary phytoestrogens. Br. J. Pharmacol..

[5] Zaineddin A.K., Buck K., Vrieling A., Heinz J., Flesch-Janys D., Linseisen J., Chang-Claude J. (2012). The association between dietary lignans, phytoestrogen-rich foods, and fiber intake and postmenopausal breast cancer risk: A German case-control study. Nutr. Cancer.

[6] Peterson J., Dwyer J., Adlercreutz H., Scalbert A., Jacques P., McCullough M.L. (2010). Dietary lignans: Physiology and potential for cardiovascular disease risk reduction. Nutr. Rev..

[7] Rothwell J.A., Perez-Jimenez J., Neveu V., Medina-Remón A., M’Hiri N., García-Lobato P., Manach C., Knox C., Eisner R., Wishart D.S. (2013). Phenol-Explorer 3.0: A major update of the Phenol-Explorer database to incorporate data on the effects of food processing on polyphenol content. Database.

[8] Landete J.M. (2012). Plant and mammalian lignans: A review of source, intake, metabolism, intestinal bacteria and health. Food Res. Int..

[9] Miles F.L., Navarro S.L., Schwarz Y., Gu H., Djukovic D., Randolph T.W., Shojaie A., Kratz M., Hullar M.A.J., Lampe P.D. (2017). Plasma metabolite abundances are associated with urinary enterolactone excretion in healthy participants on controlled diets. Food Funct..

[10] Fuentealba C., Figuerola F., Estévez A.M., Basatías J.M., Munoz O. (2014). Bioaccessibility of lignans from flaxseed (*Linum usitatissimum* L.) determined by single-batch in vitro simulation of the digestive process. J. Sci. Food Agric..

[11] Setchell K.D.R., Brown N.M., Zimmer-Nechemias L., Wolfe B., Jha P., Heubi J.E. (2014). Metabolism of secoisolariciresinol-diglycoside the dietary precursor to the intestinally derived lignan enterolactone in humans. Food Funct..

[12] Teponno R.B., Kusari S., Spiteller M. (2016). Recent advances in research on lignans and neolignans. Nat. Prod. Rep..

[13] Di Y., Jones J., Mansell K., Whiting S., Fowler S., Thorpe L., Billinsky J., Viveky N., Cheng P.C., Almousa A. (2017). Influence of flaxseed lignan supplementation to older adults on biochemical and functional outcome measures of inflammation. J. Am. Coll. Nutr..

[14] Bolvig A.K., Adlercreutz H., Theil P.K., Jørgensen H., Knudsen K.E.B. (2016). Absorption of plant lignans from cereals in an experimental pig model. Br. J. Nutr..

[15] Quartieri A., García-Villalba R., Amaretti A., Raimondi S., Leonardi A., Rossi M., Tomàs-Barberàn F. (2016). Detection of novel metabolites of flaxseed lignans in vitro and in vivo. Mol. Nutr. Food Res..

[16] Gaya P., Peirotén Á., Medina M., Landete J.M. (2017). Bifidobacterium adolescentis INIA P784: The first probiotic bacterium capable of producing enterodiol from lignan extracts. J. Funct. Foods.

[17] Peirotén Á., Gaya P., Álvarez I., Bravo D., Landete J.M. (2019). Influence of different lignan compounds on enterolignan production by *Bifidobacterium* and *Lactobacillus* strains. Int. J. Food Microbiol..

[18] Mosele J.I., Macià A., Motilva M.-J. (2015). Metabolic and microbial modulation of the large intestine ecosystem by non-absorbed diet phenolic compounds: A review. Molecules.

[19] Liu Z., Saarinen N.M., Thompson L.U. (2006). Sesamin is one of the major precursors of mammalian lignans in sesame seed (*Sesamum indicum*) as observed in vitro and in rats. J. Nutr..

[20] Corona G., Kreimes A., Barone M., Turroni S., Brigidi P., Keleszade E., Costabile A. (2020). Impact of lignans in oilseed mix on gut microbiome composition and enterolignan production in younger healthy and premenopausal women: An in vitro pilot study. Microb. Cell Factories.

[21] Struijs K., Vincken J.-P., Gruppen H. (2009). Bacterial conversion of secoisolariciresinol and anhydrosecoisolariciresinol. J. Appl. Microbiol..

[22] Kezimana P., Dmitriev A.A., Kudryavtseva A.V., Romanova E.V., Melnikova N.V. (2018). Secoisolariciresinol diglucoside of flaxseed and its metabolites: Biosynthesis and potential for nutraceuticals. Front. Genet..

[23] Kuo H.-J., Wei Z.-Y., Lu P.-C., Huang P.-L., Lee K.-T. (2014). Bioconversion of pinoresinol into matairesinol by use of recombinant *Escherichia coli*. Appl. Environ. Microbiol..

[24] Smeds A., Saarinen N.M., Hurmerinta T.T., Penttinen P.E., Sjöholm R.E., Mäkelä S.I. (2004). Urinary excretion of lignans after administration of isolated plant lignans to rats: The effect of single dose and ten-day exposures. J. Chromatogr. B.

[25] Lærke H.N., Mortensen M.A., Hedemann M.S., Knudsen K.E.B., Penalvo J.L., Adlercreutz H. (2009). Quantitative aspects of the metabolism of lignans in pigs fed fibre-enriched rye and wheat bread. Br. J. Nutr..

[26] Lagkouvardos I., Kläring K., Heinzmann S.S., Platz S., Scholz B., Engel K.-H., Schmitt-Kopplin P., Haller D., Rohn S., Skurk T. (2015). Gut metabolites and bacterial community networks during a pilot intervention study with flaxseeds in healthy adult men. Mol. Nutr. Food Res..

[27] Nurmi T., Mursu J., Peñalvo J.L., Poulsen H.E., Voutilainen S. (2010). Dietary intake and urinary excretion of lignans in Finnish men. Br. J. Nutr..

[28] Milder I.E.J., Kuijsten A., Arts I.C.W., Feskens E.J.M., Kampman E., Hollman P.C.H., Veer P.V. (2007). Relation between plasma enterodiol and enterolactone and dietary intake of lignans in a Dutch endoscopy-based population. J. Nutr..

[29] Eeckhaut E., Struijs K., Possemiers S., Vincken J.-P., De Keukeleire D., Verstraete W. (2008). Metabolism of the lignan macromolecule into enterolignans in the gastrointestinal lumen as determined in the simulator of the human intestinal microbial ecosystem. J. Agric. Food Chem..

[30] Chang H., Yao S., Tritchler D., Hullar M.A., Lampe J.W., Thompson L.U., McCann S.E. (2019). Genetic variation in steroid and xenobiotic metabolizing pathways and enterolactone excretion before and after flaxseed intervention in African American and European American women. Cancer Epidemiol. Biomarkers Prev..

[31] Kuijsten A., Arts I.C.W., Veer P.V., Hollman P.C.H. (2005). The relative bioavailability of enterolignans in humans is enhanced by milling and crushing of flaxseed. J. Nutr..

[32] Hålldin E., Eriksen A.K., Brunius C., Da Silva A.B., Bronze M., Hanhineva K., Aura A.-M., Landberg R. (2019). Factors explaining interpersonal variation in plasma enterolactone concentrations in humans. Mol. Nutr. Food Res..

[33] Brito A.F., Zang Y. (2019). A review of lignan metabolism, milk enterolactone concentration, and antioxidant status of dairy cows fed flaxseed. Molecules.

[34] Johnson S.L., Kirk R.D., DaSilva N.A., Ma H., Seeram N.P., Bertin M.J. (2019). Polyphenol microbial metabolites exhibit gut and blood–brain barrier permeability and protect murine microglia against LPS-induced inflammation. Metabolites.

[35] Possemiers S., Bolca S., Eeckhaut E., Depypere H., Verstraete W. (2007). Metabolism of isoflavones, lignans and prenylflavonoids by intestinal bacteria: Producer phenotyping and relation with intestinal community. FEMS Microbiol. Ecol..

[36] Knudsen K.E.B., Serena A., Kjaer A.K.B., Tetens I., Heinonen S.-M., Nurmi T., Adlercreutz H. (2003). Rye bread in the diet of pigs enhances the formation of enterolactone and increases its levels in plasma, urine and feces. J. Nutr..

[37] Heinonen S., Nurmi T., Liukkonen K., Poutanen K., Wähälä K., Deyama T., Nishibe S., Adlercreutz H. (2001). In vitro metabolism of plant lignans: New precursors of mammalian lignans enterolactone and enterodiol. J. Agric. Food Chem..

[38] Hullar M.A.J., Lancaster S.M., Li F., Tseng E., Beer K., Atkinson C., Wähälä K., Copeland W.K., Randolph T.W., Newton K.M. (2004). Enterolignan-producing phenotypes are associated with increased gut microbial diversity and altered composition in premenopausal women in the united states. Cancer Epidemiol. Biomarkers Prev..

[39] Knust U., Spiegelhalder B., Strowitzki T., Owen R.W. (2006). Contribution of linseed intake to urine and serum enterolignan levels in German females: A randomised controlled intervention trial. Food Chem. Toxicol..

[40] Peñalvo J.L., Heinonen S.-M., Aura A.-M., Adlercreutz H. (2005). Dietary sesamin is converted to enterolactone in humans. J. Nutr..

[41] Saarinen N.M., Smeds A., Mäkelä S.I., Ämmälä J., Hakala K., Pihlava J.M., Ryhänen E.L., Sjöholm R., Santti R. (2002). Structural determinants of plant lignans for the formation of enterolactone in vivo. J. Chromatogr. B.

[42] Klingbeil E., De La Serre C.B. (2018). Microbiota modulation by eating patterns and diet composition: Impact on food intake. Am. J. Physiol. Regul. Integr. Comp. Physiol..

[43] Wang X.-Q., Zhang A.-H., Miao J.-H., Sun H., Yan G.-L., Wu F.-F., Wang X.-J. (2018). Gut microbiota as important modulator of metabolism in health and disease. RSC Adv..

[44] Nishida A., Inoue R., Inatomi O., Bamba S., Naito Y., Andoh A. (2018). Gut microbiota in the pathogenesis of inflammatory bowel disease. Clin. J. Gastroenterol..

[45] Hjorth M.F., Roager H.M., Larsen T.M., Poulsen S.K., Licht T.R., Bahl M.I., Zohar Y., Astrup A. (2018). Pre-treatment microbial Prevotella-to-Bacteroides ratio, determines body fat loss success during a 6-month randomized controlled diet intervention. Int. J. Obes..

[46] Christensen L., Roager H.M., Astrup A., Hjorth M.F. (2018). Microbial enterotypes in personalized nutrition and obesity management. Am. J. Clin. Nutr..

[47] Schnorr S.L., Candela M., Rampelli S., Centanni M., Consolandi C., Basaglia G., Turroni S., Biagi E., Peano C., Severgnini M. (2014). Gut microbiome of the Hadza hunter-gatherers. Nat. Commun..

[48] De Filippo C., Cavalieri D., Di Paola M., Ramazzotti M., Poullet J.B., Massart S., Collini S., Pieraccini G., Lionetti P. (2010). Impact of diet in shaping gut microbiota revealed by a comparative study in children from Europe and rural Africa. Proc. Natl Acad. Sci. USA.

[49] Bess E.N., Bisanz J.E., Yarza F., Bustion A.E., Rich B.E., Li X., Kitamura S., Waligurski E., Ang Q.Y., Alba D.L. (2020). Genetic basis for the cooperative bioactivation of plant lignans by *Eggerthella lenta* and other human gut bacteria. Nat. Microbiol..

[50] Clavel T., Doré J., Blaut M. (2006). Bioavailability of lignans in human subjects. Nutr. Res. Rev..

[51] Nose M., Fujimoto T., Takeda T., Nishibe S., Ogihara Y. (1992). Structural transformation of lignan compounds in rat gastrointestinal tract. Planta Med..

[52] Patel D., Vaghasiya J., Pancholi S.S., Paul A. (2012). Therapeutic potential of secoisolariciresinol diglucoside: A plant lignan. Int. J. Pharm. Sci. Drug Res..

[53] Wang L.-Q., Meselhy M.R., Li Y., Qin G.-W., Hattori M. (2000). Human intestinal bacteria capable of transforming secoisolariciresinol diglucoside to mammalian lignans, enterodiol and enterolactone. Chem. Pharm. Bull..

[54] Studenik S., Vogel M., Diekert G. (2012). Characterization of an O-demethylase of *Desulfitobacterium hafniense* DCB-2. J. Bacteriol..

[55] Engelmann T., Kaufmann F., Diekert G. (2001). Isolation and characterization of a veratrol:corrinoid protein methyl transferase from *Acetobacterium dehalogenans*. Arch. Microbiol..

[56] Kaufmann F., Wohlfarth G., Diekert G. (1997). Isolation of O-demethylase, an ether-cleaving enzyme system of the homoacetogenic strain MC. Arch. Microbiol..

[57] Naidu D., Ragsdale S.W. (2001). Characterization of a three-component vanillate O-demethylase from *Moorella thermoacetica*. J. Bacteriol..

[58] Chen J.-X., Deng C.-Y., Zhang Y.-T., Liu Z.-M., Wang P.-Z., Liu S.-L., Qian W., Yang D.-H. (2016). Cloning, expression, and characterization of a four-component O-demethylase from human intestinal bacterium *Eubacterium limosum* ZL-II. Appl. Microbiol. Biotechnol..

[59] Gaya P., Medina M., Sánchez-Jiménez A., Landete J.M. (2016). Phytoestrogen metabolism by adult human gut microbiota. Molecules.

[60] Woting A., Clavel T., Loh G., Blaut M. (2010). Bacterial transformation of dietary lignans in gnotobiotic rats. FEMS Microbiol. Ecol..

[61] Figueira I., Garcia G., Pimpão R.C., Terrasso A.P., Costa I., Almeida A.F., Tavares L., Pais T.F., Pinto P., Ventura M.R. (2017). Polyphenols journey through blood-brain barrier towards neuronal protection. Sci. Rep..

[62] Liu K., Luo M., Wei S. (2019). The bioprotective effects of polyphenols on metabolic syndrome against oxidative stress: Evidences and perspectives. Oxidative Med. Cell. Longev..

[63] Reddy V.P., Aryal P., Robinson S., Rafiu R., Obrenovich M., Perry G. (2020). Polyphenols in Alzheimer’s disease and in the gut-brain axis. Microorganisms.

[64] Crispi S., Filosa S., Di Meo F. (2018). Polyphenols-gut microbiota interplay and brain neuromodulation. Neural Regen. Res..

[65] Witkowska A.M., Waśkiewicz A., Zujko M.E., Szcześniewska D., Stepaniak U., Pająk A., Drygas W. (2018). Are total and individual dietary lignans related to cardiovascular disease and its risk factors in postmenopausal women? A nationwide study. Nutrients.

[66] Gong L., Cao W., Chi H., Wang J., Zhang H., Liu J., Sun B. (2018). Whole cereal grains and potential health effects: Involvement of the gut microbiota. Food Res. Int..

[67] Creus-Cuadros A., Tresserra-Rimbau A., Quifer-Rada P., Martínez-González M.A., Corella D., Salas-Salvadó J., Fitó M., Estruch R., Gómez-Gracia E., Lapetra J. (2017). Associations between both lignan and yogurt consumption and cardiovascular risk parameters in an elderly population: Observations from a cross-sectional approach in the PREDIMED study. J. Acad. Nutr. Diet..

[68] Song Y., Shan B., Zeng S., Zhang J., Jin C., Liao Z., Wang T., Zeng Q., He H., Wei F. (2020). Raw and wine processed *Schisandra chinensis* attenuate anxiety like behavior via modulating gut microbiota and lipid metabolism pathway. J. Ethnopharmacol..

[69] Soleymani S., Habtemariam S., Rahimi R., Nabavi S.M. (2020). The what and who of dietary lignans in human health: Special focus on prooxidant and antioxidant effects. Trends Food Sci. Technol..

[70] Serra D., Almeida L.M., Dinis T.C.P. (2018). Dietary polyphenols: A novel strategy to modulate microbiota-gut-brain axis. Trends Food Sci. Technol..

[71] Das M., Devi K.P. (2019). A mini review on the protective effect of lignans for the treatment of neurodegenerative disorders. J. Nutr. Food Lipid Sci..

[72] Bondonno C.P., Yang X., Croft K.D., Considine M.J., Ward N.C., Rich L., Puddey I.B., Swinny E., Mubarak A., Hodgson J.M. (2012). Flavonoid-rich apples and nitrate-rich spinach augment nitric oxide status and improve endothelial function in healthy men and women: A randomized controlled trial. Free Radic. Biol. Med..

[73] Laranjinha J., Santos R.M., Lourenço C.F., Ledo A., Barbosa R.M. (2012). Nitric oxide signaling in the brain: Translation of dynamics into respiration control and neurovascular coupling. Ann. N. Y. Acad. Sci..

[74] Ma Q., Xing C., Long W., Wang H.Y., Liu Q., Wang R.-F. (2019). Impact of microbiota on central nervous system and neurological diseases: The gut-brain axis. J. Neuroinflammation.

[75] Smith P.A. (2015). The tantalizing links between gut microbes and the brain. Nature.

[76] Sharon G., Sampson T.R., Geschwind D.H., Mazmanian S.K. (2016). The central nervous system and the gut microbiome. Cell.

[77] Cussotto S., Sandhu K.V., Dinan T.G., Cryan J.F. (2018). The neuroendocrinology of the microbiota-gut-brain axis: A behavioral perspective. Front. Neuroendocrinol..

[78] Forsythe P., Bienenstock J., Kunze W.A. (2014). Vagal pathways for microbiome-brain-gut axis communication. Adv. Exp. Med. Biol..

[79] Bonaz B., Sinniger V., Pellissier S. (2017). The vagus nerve in the neuro-immune axis: Implications in the pathology of the gastrointestinal tract. Front. Immunol..

[80] Bonaz B., Sinniger V., Pellissier S. (2017). Vagus nerve stimulation: A new promising therapeutic tool in inflammatory bowel disease. J. Intern. Med..

[81] Travagli R.A., Anselmi L. (2016). Vagal neurocircuitry and its influence on gastric motility. Nat. Rev. Gastroenterol. Hepatol..

[82] Rieder R., Wisniewski P.J., Alderman B.L., Campbell S.C. (2017). Microbes and mental health: A review. Brain Behav. Immun..

[83] Farzi A., Fröhlich E.E., Holzer P. (2018). Gut microbiota and the neuroendocrine system. Neurotherapeutics.

[84] Mohajeri M.H., La Fata G., Steinert R.E., Weber P. (2018). Relationship between the gut microbiome and brain function. Nutr. Rev..

[85] Cani P.D. (2016). Interactions between gut microbes and host cells control gut barrier and metabolism. Int. J. Obes. Suppl..

[86] Foster J.A., Rinaman L., Cryan J.F. (2017). Stress & the gut-brain axis: Regulation by the microbiome. Neurobiol. Stress.

[87] Martin C.R., Osadchiy V., Kalani A., Mayer E.A. (2018). The brain-gut-microbiome axis. Cell. Mol. Gastroenterol. Hepatol..

[88] Carabotti M., Scirocco A., Maselli M.A., Severi C. (2015). The gut-brain axis: Interactions between enteric microbiota, central and enteric nervous systems. Ann. Gastroenterol..

[89] Saulnier D.M., Ringel Y., Heyman M.B., Foster J.A., Bercik P., Shulman R.J., Versalovic J., Verdu E.F., Dinan T.G., Hecht G. (2013). The intestinal microbiome, probiotics and prebiotics in neurogastroenterology. Gut Microbes..

[90] Strandwitz P. (2018). Neurotransmitter modulation by the gut microbiota. Brain Res..

[91] Holzer P., Farzi A. (2014). Neuropeptides and the microbiota-gut-brain axis. Adv. Exp. Med. Biol..

[92] Cryan J.F., Dinan T.G. (2012). Mind-altering microorganisms: The impact of the gut microbiota on brain and behaviour. Nat. Rev. Neurosci..

[93] Mittal R., Debs L.H., Patel A.P., Nguyen D., Patel K., O’Connor G., Grati M., Mittal J., Yan D., Eshraghi A.A. (2017). Neurotransmitters: The critical modulators regulating gut-brain axis. J. Cell. Physiol..

[94] Zhu Y., Kawaguchi K., Kiyama R. (2017). Differential and directional estrogenic signaling pathways induced by enterolignans and their precursors. PLoS ONE.

[95] Jin J.S., Hattori M. (2009). Further studies on a human intestinal bacterium *Ruminococcus* sp. END-1 for transformation of plant lignans to mammalian lignans. J. Agric. Food Chem..

[96] Parikh M., Maddaford T.G., Austria J.A., Aliani M., Netticadan T., Pierce G.N. (2019). Dietary flaxseed as a strategy for improving human health. Nutrients.

[97] Liu H., Liu J., Wang S., Zeng Z., Li T., Liu Y., Mastriani E., Li Q.-H., Bao H.-X., Zhou Y.-J. (2017). Enterolactone has stronger effects than enterodiol on ovarian cancer. J. Ovarian Res..

[98] Ezzat S.M., Shouman S.A., ElKhoely A., Attia Y.M., Elsesy M.E., El Senousy A.S., Choucry M.A., El Gayed S.H., El Sayed A.A., Sattar E.A. (2018). Anticancer potentiality of lignan rich fraction of six Flaxseed cultivars. Sci. Rep..

[99] Chen L.-H., Fang J., Li H., Demark-Wahnefried W., Lin X. (2007). Enterolactone induces apoptosis in human prostate carcinoma LNCaP cells via a mitochondrial-mediated, caspase-dependent pathway. Mol. Cancer Ther..

[100] Köse L., Gülçin İ. (2017). Inhibition effects of some lignans on carbonic anhydrase, acetylcholinesterase and butyrylcholinesterase enzymes. Rec. Nat. Prod..

[101] Cuong T.D., Hung T.M., Han H.-Y., Roh H.S., Seok J.-H., Lee J.K., Jeong J.Y., Choi J.S., Kim J.A., Min B.S. (2014). Potent acetylcholinesterase inhibitory compounds from *Myristica fragrans*. Nat. Prod. Commun..

[102] Wei M., Liu Y., Pi Z., Li S., Hu M., He Y., Yue K., Liu T., Liu Z., Song F. (2019). Systematically characterize the anti-alzheimer’s disease mechanism of lignans from *S. chinensis* based on in-vivo ingredient analysis and target-network pharmacology strategy by UHPLC⁻Q-TOF-MS. Molecules.

[103] Giuliano C., Siani F., Mus L., Ghezzi C., Cerri S., Pacchetti B., Bigogno C., Blandini F. (2020). Neuroprotective effects of lignan 7-hydroxymatairesinol (HMR/lignan) in a rodent model of Parkinson’s disease. Nutrition.

[104] Espín J.C., González-Sarrías A., Tomás-Barberán F.A. (2017). The gut microbiota: A key factor in the therapeutic effects of (poly)phenols. Biochem. Pharmacol..

